# Heterogeneity of Neutrophils and Immunological Function in Neonatal Sepsis: Analysis of Molecular Subtypes Based on Hypoxia–Glycolysis–Lactylation

**DOI:** 10.1155/mi/5790261

**Published:** 2025-03-26

**Authors:** Huabin Wang, Ru Yang, Nan Chen, Xiang Li

**Affiliations:** ^1^Department of Pediatrics, Affiliated Hospital of Jining Medical University, Jining Medical University, Jining, China; ^2^Jining Key Laboratory for Prevention and Treatment of Severe Infection in Children, Affiliated Hospital of Jining Medical University, Jining, China; ^3^Shandong Provincial Key Medical and Health Discipline of Pediatric Internal Medicine, Affiliated Hospital of Jining Medical University, Jining, China; ^4^Department of Graduate Education, Kunming Medical University, Kunming, China; ^5^Department of General Practice, The Second Affiliated Hospital of Guangxi Medical University, Guangxi Medical University, Nanning, China

## Abstract

**Objective:** Hypoxia–glycolysis–lactylation (HGL) may play a crucial role in neonatal sepsis (NS). This study aims to identify HGL marker genes in NS and explore immune microenvironment among NS subtypes.

**Materials and Methods:** The gene expression dataset GSE69686, comprising 64 NS cases and 85 controls, was selected for analysis. Based on the screened HGL-related marker genes, diagnostic prediction models were constructed using nine machine learning algorithms, and molecular subtypes of NS were identified through consensus clustering. Subsequently, the heterogeneity of biological functions and immune cell infiltration among the different subtypes was analyzed. Finally, the marker genes and lactylation were validated using the GSE25504 dataset, clinical samples, and mouse neutrophil, respectively.

**Results:** MERTK, HK3, PGK1, and STAT3 were identified and validated as marker genes, and the diagnostic prediction model for NS constructed using the support vector machine (SVM) algorithm exhibited optimal predictive performance. Based on gene expression patterns, two distinct NS subtypes were identified. Functional enrichment analysis highlighted significant immune-related pathways, while immune infiltration analysis revealed differences in neutrophil proportions between the subtypes. Furthermore, the expression levels of marker genes were positively correlated with neutrophil infiltration. Importantly, the experimental validation results were consistent with the findings from the bioinformatics analysis.

**Conclusion:** This study identified the distinct NS subtypes and their associated marker genes. These findings will contribute to elucidating the disease's heterogeneity and establishing appropriate personalized therapeutic approaches.

## 1. Introduction

Sepsis is a life-threatening organ dysfunction caused by dysregulated host response to infection [1]. Neonatal sepsis (NS) is a systemic infectious disease in newborns in which pathogenic or conditionally pathogenic bacteria invade the bloodstream and produce toxins [2]. NS is characterized by high incidence and mortality rates and poses a significant threat to the health and survival of newborns [3–5]. Owing to their immature immune systems, newborns are more susceptible to sepsis, and the clinical manifestations of the disease are often subtle in its early stages, making early diagnosis of NS challenging. Currently, inflammatory markers such as C-reactive protein (CRP), procalcitonin (PCT), and interleukin-6 (IL-6) are used to aid in the diagnosis and prediction of NS [6, 7]; however, there is still room for improvement in their sensitivity and specificity [8, 9]. Therefore, it is imperative to explore the molecular changes involved in the pathogenesis of NS and identify novel and effective biomarkers that can facilitate early diagnosis and treatment of NS.

Lactylation is a novel modification involving metabolic reprogramming and epigenetics [10]. During sepsis, the inflammatory responses and tissue hypoperfusion lead to tissue hypoxia, which triggers glycolysis metabolic reprogramming [11]. This metabolic alteration results in lactate accumulation and increased lactylation, forming an interconnected pathophysiological circuit [12, 13]. Clinical studies have demonstrated that patients with NS often exhibit elevated blood lactate levels, which closely correlate with disease severity and prognosis [14–16]. Moreover, tissue hypoxia and metabolic disorders are more pronounced in neonatal patients with sepsis, possibly because of their unique developmental stage and immune status [17, 18]. Studies have shown that hypoxia–glycolysis–lactylation (HGL)-related genes (HGLRGs) are crucial in various diseases [19–21]. In particular, histone lactylation has emerged as a novel epigenetic modification that links metabolism to gene regulation [22], suggesting its potential role in the pathogenesis of sepsis. However, research on the expression profiles of HGLRGs and their relationship with the immune microenvironment of patients with sepsis is limited. Therefore, exploring the molecular mechanisms of HGL involvement in the onset and progression of sepsis could aid in the early recognition of sepsis and discovering potential new therapeutic targets.

Previous studies have demonstrated that distinct neutrophil subsets in sepsis exhibit varied proinflammatory and immunosuppressive activities, encompassing senescence, antigen presentation, and reverse migration [23]. Furthermore, the heterogeneity of neutrophils correlates with the severity of sepsis [24]. Consequently, exploring the heterogeneity of neutrophils in different subsets of patients with sepsis is beneficial for personalized treatment strategies, ultimately aiming to enhance patient survival rates.

In this study, we aimed to explore the expression patterns of HGLRGs in sepsis, construct a risk prediction model based on the HGLRG expression profile, and identify the molecular subtypes. By investigating the differences in gene expression, immune cells, and inflammatory factors among the different subtypes, our findings contribute to the early diagnosis and personalized treatment of patients with NS.

## 2. Materials and Methods

### 2.1. Source of Bioinformatics Data

**Figure 1:**
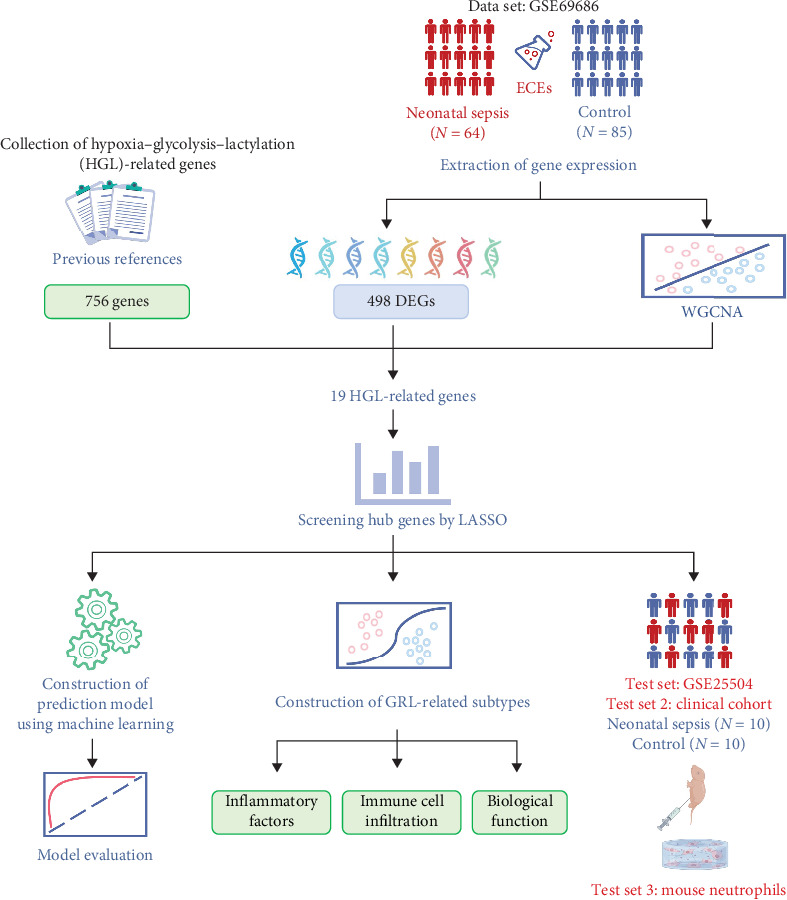
Diagram of the study workflow.

The workflow of this study is illustrated in Figure 1. Gene expression data used in this study were obtained from the Gene Expression Omnibus (GEO), a public repository maintained by the National Center for Biotechnology Information (NCBI), which provides access to microarray and sequencing data from various studies. We selected 2 NS datasets: GSE69686 (GPL20292) [25, 26], which included 64 NS cases and 85 controls, and GSE25504 (GPL6947) [27, 28], which was used as a validation set containing 28 NS cases and 35 controls. The HGL gene set, comprising 756 genes, was obtained from the published literature [20].

### 2.2. Identification of Differentially Expressed Genes (DEGs)

Using the “limma” R package within R version 4.4.1, we screened for DEGs in GSE69686. The selection criteria were set as |log2 fold change (FC)| > 0.5 and *p* < 0.05. In this analysis, genes with log FC > 0.5 and *p* < 0.05 were considered upregulated, while those with log FC < −0.5 and *p* < 0.05 were considered downregulated. Volcano plots of the DEGs were generated using the “ggplot2” R package, and heatmaps were created using the “Pheatmap” R package.

### 2.3. Identifying Key Weighted Gene Coexpression Network Analysis (WGCNA) Modules

In this study, we employed R version 4.4.1 to perform the WGCNA. The first step in the WGCNA is to select a soft threshold, a method for deriving the optimal cutoff value. The scale-free topology fit index was set to 1–10, 11, 13, 15, 17, and 19, ensuring that the constructed network followed a power-law distribution and closely resembled the actual data. Subsequently, the topological overlap and adjacency matrix (with minModuleSize set to 300 for module splitting) were constructed based on the gene expression data. A flexible, dynamic tree cutting algorithm was employed to generate a dendrogram that included distinguishable modules represented by different colors. Finally, modules significantly correlated with the phenotypic data were identified, and each module's eigengenes were determined.

### 2.4. Screening of Marker Genes

Least absolute shrinkage and selection operator (LASSO) regression is a data mining technique that applies L1-penalization (lambda) to set the coefficients of less important variables to zero, filtering out significant variables and constructing an optimal classification model. In this study, we employed R version 4.4.1 to perform the LASSO. Random numbers generated in 2024 were used in the model, and a tenfold cross-validation approach was adopted. Initially, we utilized the “glmnet” package in R to identify the intersection of DEGs, WGCNA-related modules, and HGLRGs. Subsequently, LASSO regression was employed to screen hub genes within these intersecting genes. Finally, these hub genes were validated using an external dataset, and successfully validated genes were identified as marker genes.

### 2.5. Validation of Four Marker Genes in the Clinical Cohort by Real-Time Quantitative PCR (RT-qPCR)

This study was conducted following the Declaration of Helsinki (revised in 2013) and was approved by the Ethics Committee of the Affiliated Hospital of Jining Medical University (2022C242). Consent was obtained from the guardians before the commencement of the study. According to previous studies [29–31], blood samples were collected from 10 normal controls and 10 neonates with sepsis hospitalized in the NICU of the Affiliated Hospital of Jining Medical University between January 1, 2024, and May 31, 2024. The sepsis group consisted of neonates who were diagnosed with NS [32, 33], which encompassed both clinical and definitive diagnoses. A clinical diagnosis was established when clinical abnormalities (Table S1) were present along with any one of the following criteria: at least two positive nonspecific blood tests (Table S2), cerebrospinal fluid (CSF) examination indicating changes consistent with purulent meningitis, or detection of pathogenic bacterial DNA in the blood or CSF. A definitive diagnosis is made when clinical abnormalities are accompanied by a positive blood culture or CSF (or other sterile body fluid) culture. The control group consisted of hospitalized, uninfected, clinically stable infants. The exclusion criteria included severe congenital malformations and severe perinatal asphyxia, defined as an Apgar score of 0–3 for more than 5 min, umbilical cord blood gas pH < 7.00, or both.

Peripheral blood was collected from patients on the first day of diagnosis. Total RNA was extracted from whole blood using the FastPure Cell/Tissue Total RNA Isolation Kit V2 (Vazyme), following the manufacturer's instructions. Reverse transcription was performed using HiScript III RT SuperMix for qPCR (Vazyme). RT-qPCR was performed using ChamQ Universal SYBR qPCR Master Mix (Vazyme). GAPDH was used as a reference gene. The relative expression levels were calculated using the 2^−ΔΔCT^ method.

### 2.6. Comprehensive Analysis of Multiple Classification Models to Construct Diagnostic Prediction Models

After selecting key features from all independent variables, we constructed multiple machine learning classification models. We comprehensively analyzed and compared their performances on both the training and validation sets. The specific steps were as follows:

1. Data partitioning: The samples were randomly divided into a training set and a validation set at a 7:3 ratio using the random number method in Python (0.22.1).
2. Multimodel classification analysis: Using Python libraries (scikit-learn 1.1.3, xgboost 2.0.1, and lightgbm 3.2.1), nine machine learning models were built: eXtreme Gradient Boosting (XGBoost), logistic regression, Light Gradient Boosting Machine (LightGBM), random forest, adaptive boosting (AdaBoost), decision tree, support vector machine (SVM), *k*-nearest neighbors (KNNs), and Gaussian Naive Bayes (GNB).

Subsequently, we trained and internally validated (with 10 times resampling) the aforementioned parametric models. To evaluate the discrimination ability of the models, we plotted receiver operating characteristic (ROC) curves. Calibration curves were used to assess the degree of calibration of prediction models. Additionally, decision curve analysis (DCA) curves were plotted to evaluate the clinical applicability of the models. Furthermore, precision–recall (PR) curves, which are widely used for model performance evaluations, were generated. The average precision (AP), which is the area under the PR curve, provides a valuable complement to existing model evaluation methods.

### 2.7. Consensus Clustering

Consensus clustering is a method of identifying molecular subtypes based on an estimated number of clusters. We employed the *k*-means approach in R version 4.4.1 to explore sepsis subgroups associated with the expression of key HGLRGs. In the consensus clustering analysis, the *k*-means method for subgroup classification has the advantages of good interpretability, simple implementation, and effective classification. A random seed of 123456 was set for reproducibility. The maximum number of clusters evaluated was nine, with 1000 iterations for each *k*. Euclidean distance was chosen as the clustering distance metric. The number of clusters was determined using the consensus clustering algorithm implemented in the “ConsensusClusterPlus” R package. The graphical results include a consensus clustering heatmap and consensus cumulative distribution function (CDF) plot. The consistency and stability of clustering across various *k*-values were evaluated using the visualization method of CDF plots, enabling the selection of the optimal number of clusters.

### 2.8. Analysis of Differences Among Sepsis Subtypes

Using the “limma” R package, we screened for DEGs among sepsis patient subtypes in GSE69686. The selection criteria were |log2 FC| > 0.5 and *p* < 0.05. Volcano plots of the DEGs were generated using the “ggplot2” R package, while heatmaps were created using the “Pheatmap” R package.

### 2.9. Expression of Inflammatory Factors Among Subtypes

Using the “Hmisc” R package, we performed statistical analyses on CD163, IL1R1, IL1R2, IL18R1, MMP8, and TLR8 for the two groups. Subsequently, boxplots were generated using the “ggplot2” R package.

### 2.10. Gene Ontology (GO) and Kyoto Encyclopedia of Genes and Genomes (KEGG) Functional Enrichment Analysis

This study used the ClusterProfiler package in R to conduct GO and KEGG functional enrichment analyses. These analyses evaluated gene-related biological processes (BPs), molecular functions (MFs), cellular components (CCs), and the resulting signaling pathways. The results were considered statistically significant at *p*-value < 0.05.

### 2.11. Gene Set Variation Analysis (GSVA)

GSVA is a nonparametric and unsupervised method for assessing the enrichment of gene sets in transcriptome data. We selected KEGG pathways as the gene sets and applied the GSVA algorithm using the “GSVA” R package to compute pathway scores. Subsequently, we performed differential analysis between the two groups and visualized the pathways with |log2 FC| > 0 and *p* < 0.05.

### 2.12. Analysis of Immune Cell Infiltration on Hub Genes

Using the CIBERSORT algorithm, we analyzed RNA-seq data from patients with sepsis to determine the relative proportions of the 22 immune-infiltrating cells. Pearson's correlation analysis was then conducted to compare gene expression and immune cell content. Additionally, immune cells were compared among the different sepsis subtypes.

### 2.13. Validation of Lactylation in Mouse Neutrophils

Mouse bone marrow neutrophils were isolated using the EasySep Mouse Neutrophil Enrichment Kit according to the manufacturer's instructions (StemCell Technologies). The culture medium for mouse neutrophils was RPMI 1640 supplemented with 10% FBS and 1% penicillin-streptomycin. Cells were cultured at 37°C with 5% CO_2_. The mouse neutrophils were stimulated with lipopolysaccharide (LPS) (Solarbio) for 30 min. The medium was then removed. Cellular proteins were extracted and quantified according to the procedures outlined in the instructions for cell lysis buffer for western blotting and IP (Beyotime) and the BCA Protein Assay Kit (Beyotime).

Equal amounts of lysates (20 μg) were resolved by sodium dodecyl sulfate-polyacrylamide gel electrophoresis (SDS-PAGE). Equal loading was verified by Coomassie staining and then transferred to polyvinylidene difluoride membranes (Beyotime). The membranes were subsequently blocked with 5% skim milk and incubated with primary antibodies (Pan-Kla, diluted 1:1000, PTM-Bio) overnight at 4°C. They were incubated with secondary antibodies for 1 h at 37°C. Signals were detected using the ECL kit (Epizyme).

### 2.14. Statistical Analysis

Statistical analyses were performed using R version 4.4.1. The diagnostic value of key genes was evaluated using ROC curve analysis. Student's *t*-test was used to explore and compare the results between the two groups. Statistical significance was considered when the *p*-value was less than 0.05.

## 3. Result

### 3.1. Identification of DEGs

**Figure 2:**
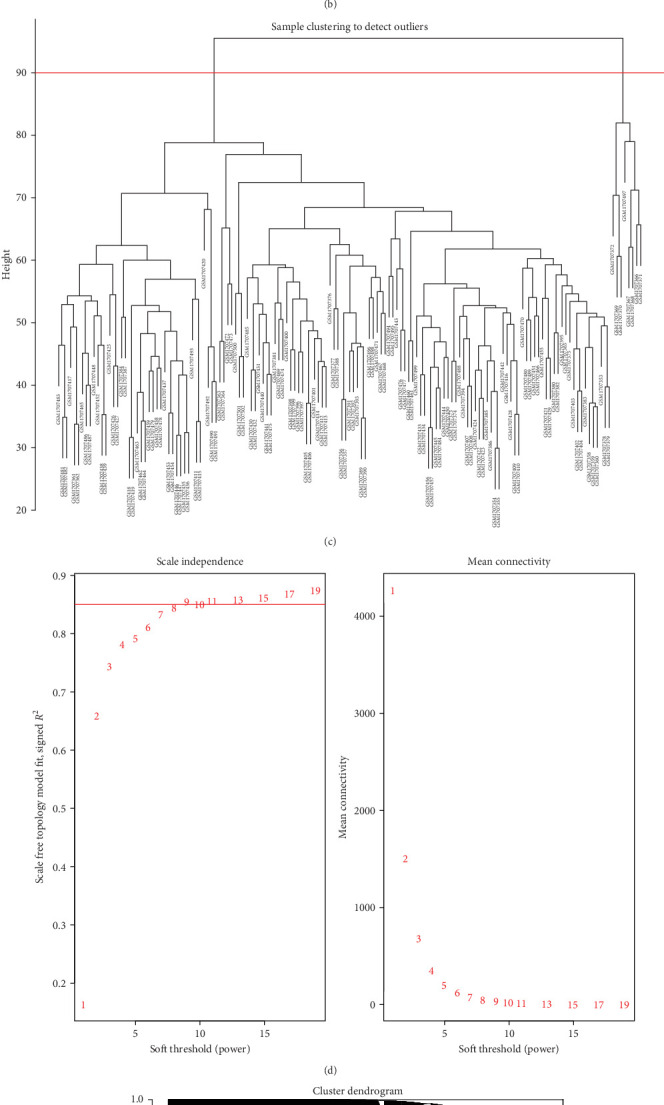
Differentially expressed gene (DEG) and weighted gene coexpression network analysis (WGCNA). (A) Volcano plot of neonatal sepsis-related DEGs in the GSE69686 dataset. The grouping in the calculation process includes a neonatal sepsis group and a normal control group. The abscissa is the log2 fold change, the ordinate is −log10 (adjusted *p*-value), yellow nodes represent upregulated DEGs, blue nodes represent downregulated DEGs, and gray nodes represent genes that are not significantly differentially expressed. (B) Heatmaps of neonatal sepsis-related DEGs in the GSE69686 datasets. The horizontal axis indicates the patient ID, the vertical axis indicates the respective DEGs, red represents high gene expression, blue represents low gene expression, blue bars indicate the control group, and red bars indicate neonatal sepsis. (C) Clustered dendrograms are cut at a height of 90 to detect and combine similar modules. (D) Filter the soft threshold powers value, the most appropriate when the screening result is 9. (E) Display and merge clustering trees. (F) Association of gene modules with clinical traits (screen the modules whose correlation is greater than or equal to 0.4, and screen out two modules in total yellow and brown).

Initially, sample data from the GSE69686 dataset were normalized, and boxplots were constructed to represent the normalized data (Figure S1). Subsequently, we utilized the “limma” R package to assess DEGs between the sepsis and normal groups within the dataset. In total, 498 DEGs were identified in the GSE69686 dataset. These DEGs were visualized using volcano plots and heat maps (Figure 2A,B).

### 3.2. Identifying Key WGCNA Modules

First, we employed WGCNA to associate each module with the corresponding clinical traits, thereby analyzing the BPs. All genes in the GSE69686 dataset were included in the WGCNA, and an appropriate soft-thresholding value was selected. The results indicated that the optimal number was 9 (Figure 2D). Subsequently, we generated and merged a clustering dendrogram (Figure 2C,E) and identified two modules (yellow and brown) with correlation coefficients greater than 0.4 (Figure 2F).

### 3.3. Screening of Marker Genes

**Figure 3:**
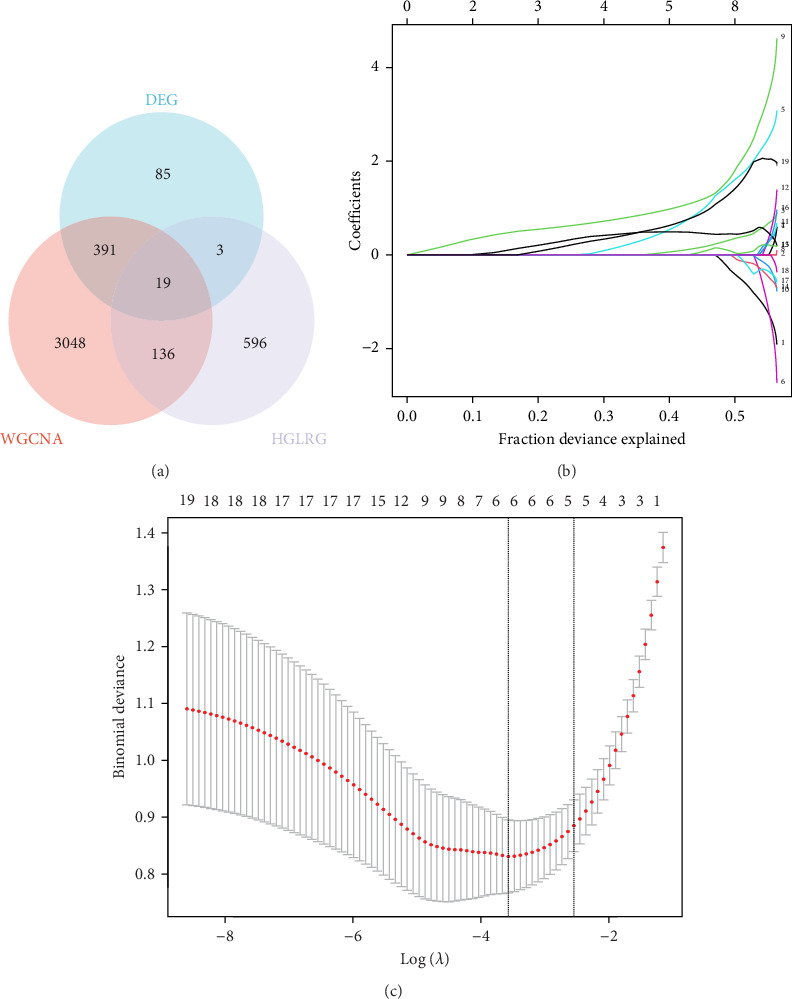
Venn diagrams and establishment of diagnostic biomarkers by LASSO regression analysis. (A) Venn diagrams of differentially expressed genes (DEGs), weighted gene coexpression network analysis (WGCNA), and hypoxia–glycolysis–lactylation (HGL)-related genes (HGLRGs). The blue circle indicates DEGs in GSE69686, the red circle indicates the genes of two modules (yellow and brown) with correlation coefficients greater than 0.4 in WGCNA, and the purple circle indicates genes related to HGL. (B) LASSO coefficient profiles of the 19 genes in neonatal sepsis. The grouping in the calculation process includes a neonatal sepsis group and a normal control group. (C) The log (lambda) sequence was used to construct a coefficient profile diagram. The LASSO model's optimal parameter (lambda) was chosen.

Through the literature review, we identified 756 genes related to HGL. By intersecting these genes with the WGCNA module genes (3594 genes) and DEGs (498 genes), we identified 19 HGLRGs (Figure 3A): HK3, PGK1, STAT3, MERTK, LDHA, EXT1, PGM2, TIPARP, PAM, S100A11, PFKFB2, GLRX, WSB1, HSDL2, PYGL, LXN, RRAGD, NUDT5, and PFKFB3.

**Figure 4:**
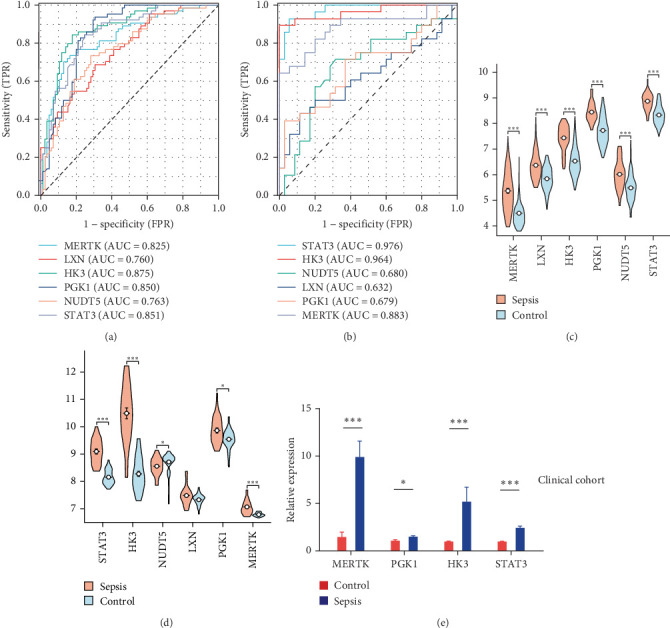
Validation of hypoxia–glycolysis–lactylation-related marker genes. (A, B) ROC showed the diagnostic performance of the hub genes in GSE69686 and GSE25504. The grouping in the calculation process includes a neonatal sepsis group and a normal control group. (C, D) The expression of hub genes between the neonatal sepsis and control group in GSE69686 and GSE25504. (E) Analysis of four marker genes in the clinical cohort by real-time quantitative PCR. AUC, area under the curve; FPR, false positive rate; TPR, true positive rate. *⁣*^*∗*^*p* < 0.05; *⁣*^*∗∗∗*^*p*  < 0.001.

Using LASSO (Figure 3B,C), we obtained six hub genes (MERTK, LXN, HK3, PGK1, NUDT5, and STAT3) from HGLRGs. We conducted a ROC analysis to evaluate the diagnostic performance of these hub genes. HK3 exhibited the highest area under the curve (AUC) value (0.875) in the training set. In the validation set, STAT3 exhibited the highest AUC (0.976) (Figure 4A,B). In the validation set, four genes (MERTK, LXN, PGK1, and STAT3) showed significantly higher expression in the NS group than in the control group (Figure 4C,D).

### 3.4. Verification of Selected Key Genes at the Transcriptional Level

RT-qPCR was used to compare the expression of the four key genes between disease and control groups in the clinical cohort (Figure 4E). The expression patterns of these genes were consistent with the trends observed in the microarray analysis.

### 3.5. Comprehensive Analysis of Multiple Classification Models to Construct Diagnostic Prediction Models

**Figure 5:**
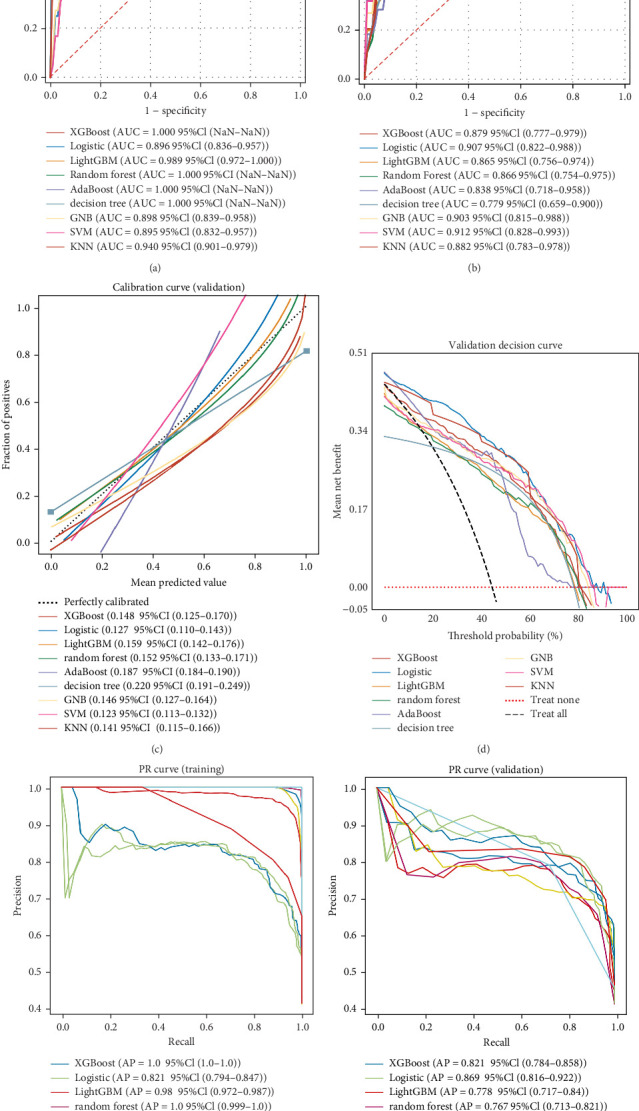
Comprehensive analysis of machine learning diagnostic prediction models. (A) Receiver operating characteristic (ROC) curve and area under the curve (AUC) for the training set, assessing the diagnostic performance of the machine learning model. (B) The ROC curve and AUC for the validation set evaluate the model's generalizability. (C) A calibration curve for the validation set demonstrates the agreement between predicted probabilities and observed frequencies of the outcome. (D) The clinical decision curve for the validation set aids clinical decision-making by showing the net benefit of using the model at different threshold probabilities. (E) Precision–recall (PR) curve and average precision (AP) value for the training set. The *y*-axis represents precision, while the *x*-axis indicates recall. These metrics provide insights into the model's ability to identify true positives while minimizing false positives. (F) PR curve and AP value for the validation set. If one model's PR curve is entirely dominated (lies below) by another model's curve, it suggests the superior performance of the latter regarding precision and recall. Such comparison aids in determining the relative merits of different models.

Nine machine learning models were trained and resampled 10 times. The results demonstrated that XGBoost, random forest, AdaBoost, and the decision trees exhibited the highest discrimination in the training set (Figure 5A). However, in the internal validation set, the SVM showed superior discrimination (Figure 5B). Although the AUC metric focuses on the predictive accuracy of models, it neither indicates their clinical usability nor determines their preference. Therefore, we evaluated the prediction models using the calibration, DCA, and PR curves. These assessments consistently indicated better performance of the SVM in the validation set (Figure 5). Upon comprehensive analysis, it was observed that XGBoost, random forest, AdaBoost, and decision tree may have suffered from overfitting. In contrast, SVM demonstrated stability and strong generalization ability, making it the optimal model.

### 3.6. Consensus Clustering and Identification of DEGs of Subtypes

**Figure 6:**
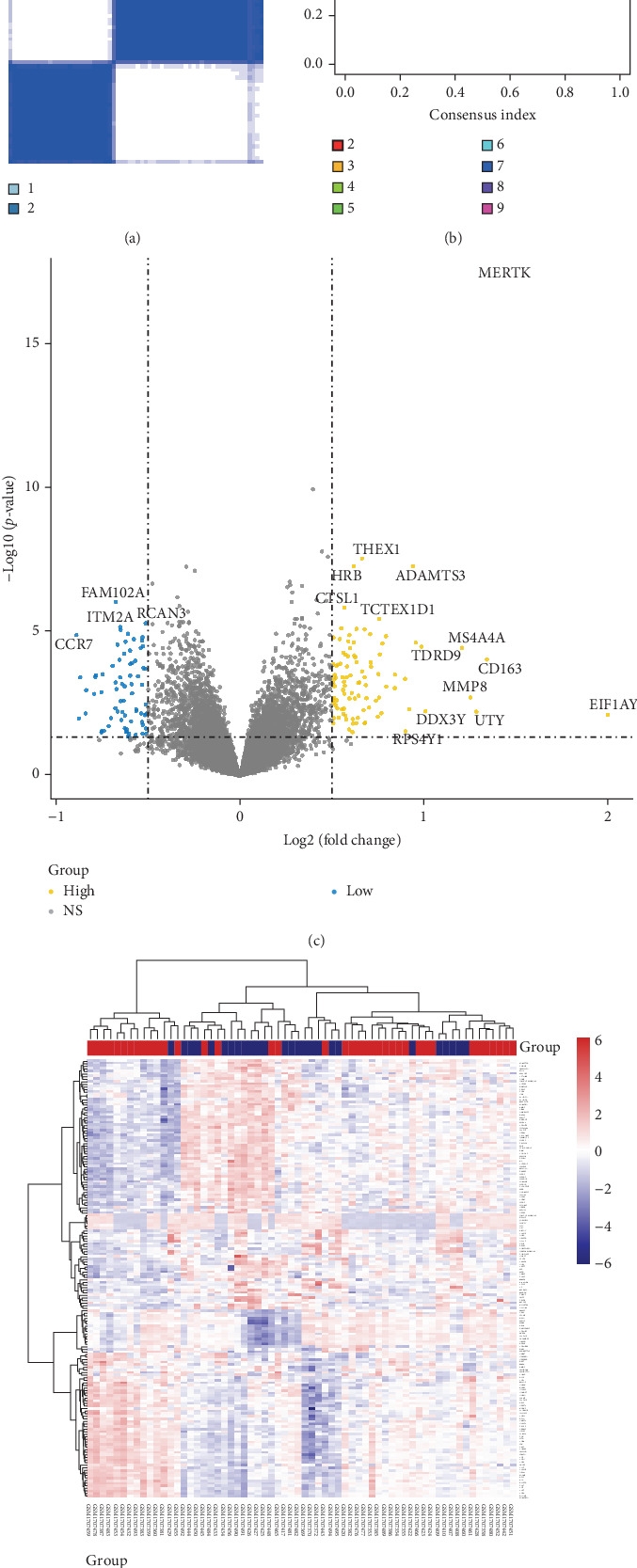
Subtype analysis of neonatal sepsis patients. (A) Sixty-four neonatal sepsis patients in the GSE69686 dataset were grouped into two clusters according to the consensus clustering matrix (*k* = 2). (B) Consensus among clusters for each category number *k*. (C) Volcano plot of the subtype analysis of neonatal sepsis in the GSE69686 datasets. The grouping in the calculation process includes subgroup 1 and subgroup 2 of neonatal sepsis. (D) Heatmaps of the subgroup analysis of neonatal sepsis in the GSE69686 datasets. CDF, cumulative distribution function.

Consistency clustering analysis revealed that clustering patients with sepsis based on key genes related to HGL yielded optimal results when divided into two groups (Figure 6A,B). Subsequently, we employed the “limma” package to identify DEGs between sepsis subtypes, identifying 170 DEGs. These DEGs were visualized using volcano plots and heat maps (Figure 6C,D).

### 3.7. Functional Enrichment Analysis

**Figure 7:**
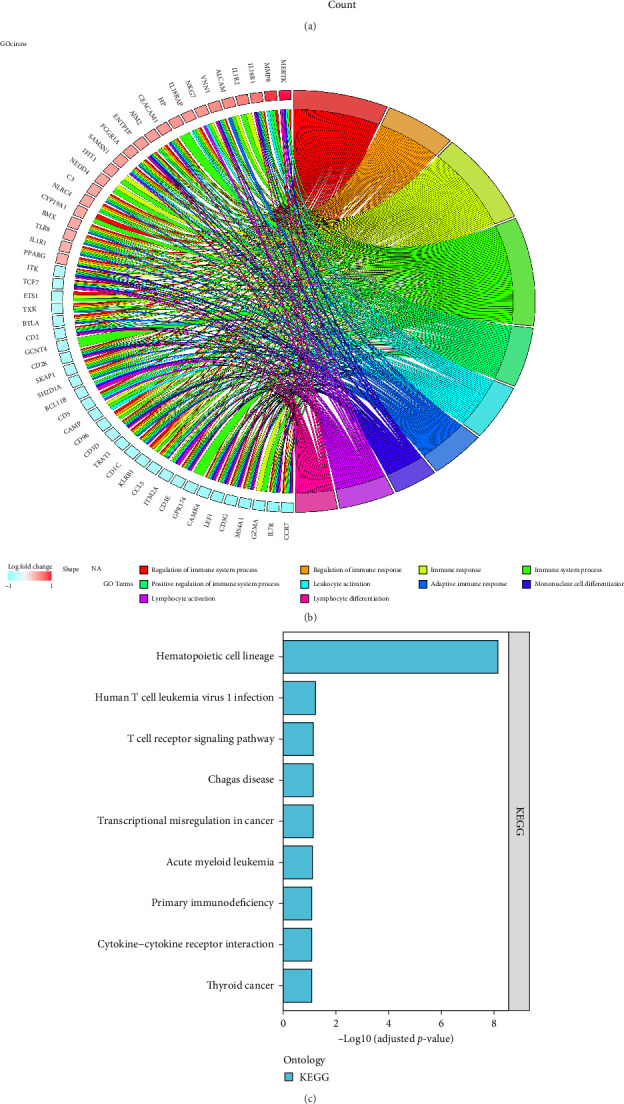
Heterogeneity in biological functions between subtypes. (A) Gene Ontology (GO) enrichment analysis divides gene functions into three categories: biological processes (BPs), cellular components (CCs), and molecular functions (MFs). (B) The first 10 items of the BP categories are displayed. (C) The pathways of Kyoto Encyclopedia of Genes and Genomes (KEGG) pathway enrichment analysis. (D) Histogram of the differential pathway of gene set variation analysis (GSVA) enrichment analyses.

Functional enrichment analysis of the 170 DEGs was performed using GO and KEGG annotations. In the BP assessment, DEGs were mostly engaged in regulating the immune system process, immune response, positive regulation of immune system processes, and other BPs. In the CC assessment, the DEGs were mostly engaged on the external side of the plasma membrane, cell surface, side of the membrane, specific granule lumen, specific granule, and other CCs. In the MF assessment, DEGs were mostly engaged in IL-1 receptor activity, cytokine receptor activity, immune receptor activity, signaling receptor activity, molecular transducer activity, and other MFs (Figure 7A,B). According to the KEGG analysis, DEGs were particularly abundant in the hematopoietic cell lineage, human T cell leukemia virus 1 infection, and other pathways (Figure 7C).

After conducting pathway enrichment analysis using GSVA, we performed differential analysis between the two subtypes and identified 24 significantly different pathways, including primary immunodeficiency, ribosomes, basal transcription factors, proteasomes, hematopoietic cell lineage, autophagy regulation, and others (Figure 7D).

### 3.8. Heterogeneity of Immune Cell Infiltration and Inflammation Levels Between HGL Subtypes

**Figure 8:**
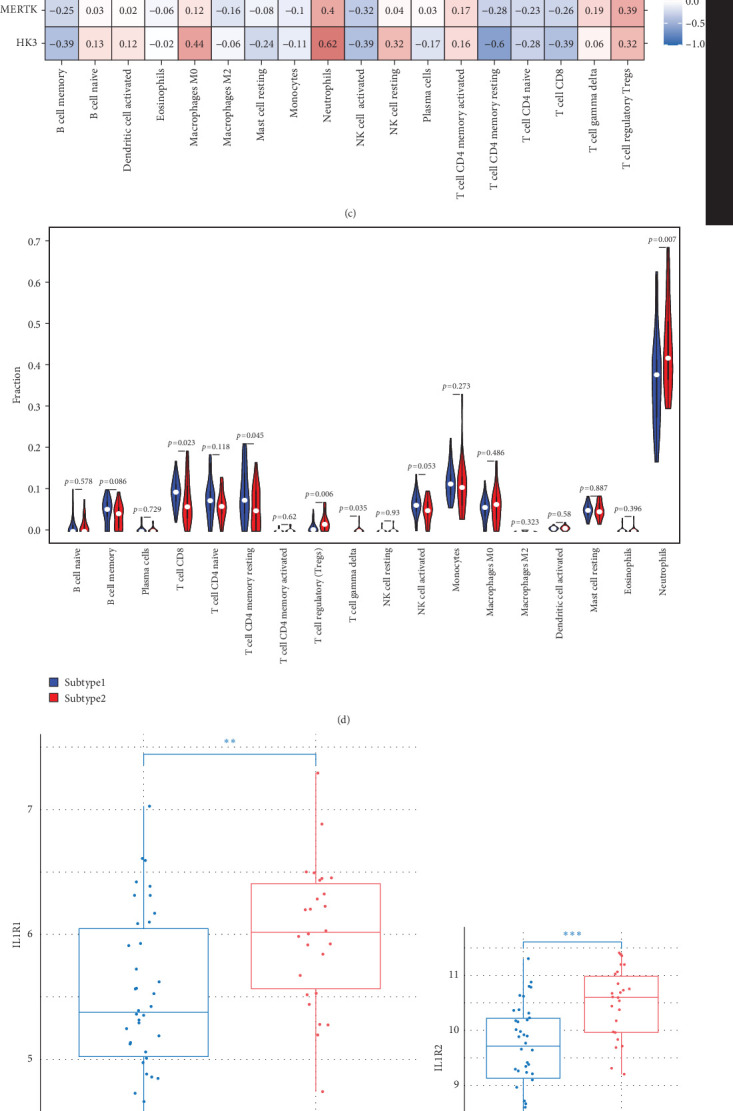
The immune cell infiltration is associated with signature genes and the expression of the immune factor. (A) The proportion of immune cells in neonatal sepsis. (B) Heatmap of the correlation between immune cells. (C) The association between hub genes and immune cell infiltration. Red represents a positive correlation, while blue represents a negative correlation. (D) The violin plot shows the immune cell infiltration between subtype1 and subtype2. (E–J) The expression of CD163, IL1R1, IL1R2, IL18R1, MMP8, and TLR8 in two subtypes.

We analyzed their expression concerning immune cell dynamics to further elucidate the potential molecular mechanisms by which the hub genes influence NS progression. Our results show the proportions of immune cells and their Pearson correlations in patients with NS (Figure 8A,B). Notably, marker genes were significantly correlated with multiple immune cell types, particularly neutrophils (Figure 8C). Additionally, we observed variations in the proportions of immune cells (neutrophils, T cell gamma delta, T cell regulatory, T cell CD4 memory resting, and T cell CD8) between the two NS subtypes (Figure 8D).

We compared the inflammatory factors between the two NS subgroups and found that CD163, IL1R1, IL1R2, IL18R1, MMP8, and TLR8 exhibited statistically significant differences in expression between the two groups (Figure 8E–J).

### 3.9. Validation of Lactylation in Mouse Neutrophils

**Figure 9:**
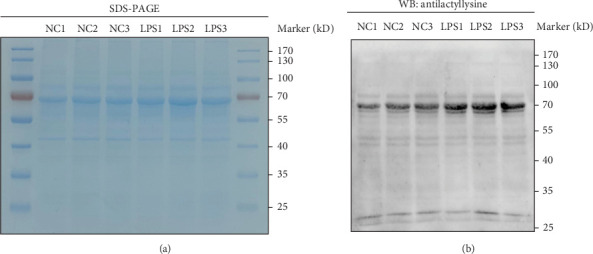
In mouse neutrophils from the LPS group, the level of lactylation was significantly higher than that in the control group. (A) The gel was stained with Coomassie blue. (B) Western blot analysis determined lactylation levels in mouse neutrophils.

Western blot analysis compared the lactylation levels in mouse neutrophils between the control and LPS groups. Consistent protein loading was ensured for each sample (Figure 9A), and the results indicated a significant increase in lactylation in the LPS group (Figure 9B).

## 4. Discussion

NS is characterized by cryptic onset, high incidence, and high mortality rate [3]. Recent research indicates that lactylation is closely associated with the development and progression of sepsis [12]. During sepsis, impaired microcirculation and an insufficient oxygen supply can produce large amounts of lactate [34]. However, there are few studies on the mechanisms of action of HGLRGs in the pathophysiology of sepsis. Therefore, there is an urgent need to explore the potential biomarkers related to HGL to optimize the diagnosis and treatment of NS.

This study identified six core genes related to HGL through WGCNA and LASSO regression. Four marker genes were successfully validated using external datasets and clinical samples, and all four marker genes exhibited higher expression in the sepsis group. Furthermore, we developed predictive models using machine learning techniques demonstrating good predictive performance. These findings suggest that HGL-related marker genes may serve as molecular biomarkers for the early diagnosis of NS.

MERTK is a gene encoding a transmembrane protein with a tyrosine kinase domain. Previous studies have demonstrated increased MERTK expression in the immune cells of patients with septic shock, and persistent overexpression of MERTK has been associated with adverse outcomes [35]. However, some data have highlighted the importance of MERTK activation in the clearance of apoptotic cells, suppression of proinflammatory cytokine production, and prevention of autoreactive lymphocyte proliferation [36–39], indicating a dual role for MERTK in the prognosis of patients with sepsis [40]. This dual role suggests that moderate activation of MERTK in the early stages of sepsis may help delay disease progression. In contrast, excessive MERTK expression may indicate dysregulation of the inflammatory response, associated with adverse outcomes. This suggests that MERTK may be a therapeutic target for interventions aimed at mitigating the severity of sepsis. However, excessive MERTK expression may indicate the dysregulation of the inflammatory response, which is associated with adverse outcomes. This highlights the complexity of targeting MERTK therapeutically because insufficient and excessive activation can have detrimental effects. The implications for therapeutic targeting of MERTK include the need for precise control of its activation levels. Dynamic monitoring of MERTK changes is crucial to ensure that interventions are timed appropriately to enhance the beneficial effects or mitigate the detrimental effects. Future research should focus on developing therapeutic strategies that regulate MERTK activity, such as small-molecule inhibitors or activators, and identifying biomarkers to predict the optimal timing for such interventions.

HK3 encodes hexokinase 3, an important isoform of hexokinase involved in the glycolytic process and is recognized as a significant biomarker in various cancers [41–44]. Glycolysis is crucial in the immune response and inflammation [45]. In a sepsis-induced acute model, HK3 knockdown significantly inhibits cell proliferation, promotes inflammation, increases apoptosis, and blocks glycolysis [46]. HK3 has been identified as a key biomarker for pediatric sepsis and septic shock and exhibits good diagnostic value [47].

PGK1 encodes a glycolytic enzyme that catalyzes the conversion of 1,3-diphosphoglycerate into 3-phosphoglycerate. Research on PGK1 has primarily focused on oncology, with previous data indicating its involvement in tumor development and progression [48–51]. Regarding inflammation, PGK1 acts as a regulatory factor in macrophages [52], modulating the balance between proinflammatory and anti-inflammatory cytokines [53]. A study on acute lung injury revealed that PGK1 promotes M1 macrophage polarization by regulating NLRP3, thereby participating in the pathophysiology of acute lung injury [54]. However, research on PGK1 in NS is limited, and further investigations are required to elucidate its specific mechanisms.

STAT3 is a transcription factor mediating cellular responses to growth factors such as interleukins, KITLG/SCF, and LEP. Previous data suggested that STAT3 may be a key regulatory gene in the potential dysfunction induced by sepsis-associated acute respiratory distress syndrome [55]. One study has demonstrated that mitochondrial STAT3 exacerbates LPS-induced sepsis [56]. Other studies concluded that STAT3 plays a significant role in developing and progressing sepsis [57, 58].

These four core genes play crucial roles in the development and progression of sepsis-induced lung injury and potentially serve as novel therapeutic targets. Our study yielded similar findings, suggesting that future research on targeted therapies for these core genes could emerge as a new focus and significantly benefit pediatric patients with sepsis. Furthermore, these genes are associated with the HGL pathway, implying that impaired circulation and an insufficient oxygen supply may lead to enhanced glycolysis, increasing lactate production. Subsequently, lactate induces histone lactylation, ultimately promoting sepsis onset and progression.

Gene functional enrichment analysis revealed significant differences between HGL subtypes in immune regulation and hematopoietic function, providing new insights into the pathogenesis of NS. Notably, enrichment in the hematopoietic cell lineage pathway suggests that altered lactate metabolism may participate in disease progression by influencing the bone marrow hematopoietic microenvironment and immune cell development, which is consistent with the findings of bone marrow suppression and immune cell dysfunction in patients with sepsis. Additionally, CC enrichment showed differential gene localization in the plasma membrane and specific granules, combined with MF enrichment in cytokine receptor activity and IL-1 receptor activity, indicating that abnormal lactate metabolism may influence disease progression by modulating immune cell signal transduction and inflammatory factor release. These findings elucidate the potential connection between lactate metabolism and immune function and provide new therapeutic targets for targeted interventions in NS.

This study demonstrates a robust association between NS immune heterogeneity and HGLRG expression. By applying a refined classification of patients with NS based on HGLRG expression levels, we identified distinct immune profiles among the subtypes, particularly concerning neutrophils. Previous studies have also demonstrated a significant correlation between lactate levels and immune responses [59–61]. Lactate produced by aerobic glycolysis may exert immunosuppressive effects in sepsis [62], and lactate levels in patients with sepsis have been associated with prognosis [63]. Both immune hyperactivation and immunosuppression are detrimental to NS progression, with immunosuppression particularly noteworthy [64]. However, elucidating individual immune states in neonates poses significant challenges owing to their immature immune systems, the complex immune heterogeneity of NS, and unknown underlying mechanisms. Previous studies have confirmed that histone deacetylases (HDACs) influence macrophage polarization, thereby affecting the inflammatory response in atherosclerosis [65].

Additionally, research on tumor drug resistance has revealed that lactylation significantly enhances DNA repair efficiency. By inhibiting lactate metabolism, particularly through LDHA inhibitors, the lactylation of NBS1 at K388 can be effectively reduced, overcoming chemotherapy resistance, and markedly improving the prognosis of patients with cancer [66]. Identifying the two subtypes with distinct lactate-modified gene expression profiles and different immune cell levels has significant implications for precisely targeted therapy, personalized treatment, and immune modulation in NS. Future research should focus on validating the identified subtypes with distinct lactate-modified gene expression profiles and immune cell levels using larger multicenter studies to advance our understanding and treatment of NS. In addition, mechanistic studies are required to elucidate the drivers underlying these differences. Based on these insights, targeted therapies and personalized treatment approaches tailored to each subtype should be developed. Furthermore, immune modulation strategies should be explored to restore immune balance, and long-term follow-up studies should be conducted to assess their prognostic value and treatment effectiveness.

However, this study has several limitations. First, some results were based on publicly available data, which may be subject to various biases and confounding factors that are difficult to control. In addition, the sample size of the basic experiments conducted in this study was not particularly large, which may have limited the statistical power and ability to detect significant associations. Larger clinical and basic experimental studies are required to confirm the identified genes and their mechanisms of action in disease development and progression. Furthermore, although transcriptome analysis of whole blood samples revealed a strong correlation between neutrophils and lactylation, this analysis did not provide a single-cell-resolution transcriptomic landscape. This limits our understanding of the heterogeneity within the neutrophil population and the differential effects of lactylation on various neutrophil subsets. Future studies utilizing single-cell transcriptome analysis are necessary to deepen our understanding of this relationship and to identify potential subpopulations of neutrophils that are particularly responsive to lactylation. Finally, the generalizability of the findings to other populations or disease contexts remains uncertain. This study used a specific dataset and may not represent all populations or disease states. Therefore, additional studies in diverse populations and disease contexts are needed to validate these findings and determine their broader applicability.

## 5. Conclusion

In summary, this study makes several significant contributions to understanding NS. First, this is the first investigation of the HGL transcriptomic profile in patients with NS, uncovering a crucial association between HGL and this disease. Second, a risk prediction model for NS based on HGL markers demonstrated strong consistency and high clinical utility, effectively stratifying patients at risk of sepsis. Third, these findings deepen our understanding of the molecular characteristics and specific immune states within subgroups of this highly heterogeneous syndrome, offering insights into precision medicine. Given that lactylation is a dynamic and reversible posttranslational modification, therapeutic interventions targeting lactylation may emerge as novel treatment options for NS.

## Data Availability

Portions of the data generated or analyzed in this study were obtained from the GEO database. For original data and further inquiries, please contact the corresponding author directly.

## Supporting Information

Supporting Information Figure S1. Normalized box plots of the GSE69686 dataset samples and differentially expressed genes (DEGs). Table S1. Common infection-related clinical manifestations mentioned in the national guideline. Table S2. Abnormal nonspecific blood tests used in neonatal sepsis diagnosis in the national guideline.
