# Supplementary material for: Heterogeneity of Neutrophils and Immunological Function in Neonatal Sepsis: Analysis of Molecular Subtypes Based on Hypoxia–Glycolysis–Lactylation
Source: Mediators Inflamm. 2025 Mar 26;2025:5790261. doi: 10.1155/mi/5790261 (PMC11964727; doi:10.1155/mi/5790261)
Supplement: Supporting Information — Figure S1. Normalized box plots of the GSE69686 dataset samples and differentially expressed genes (DEGs). Table S1. Common infection-related clinical manifestations mentioned in the national guideline. Table S2. Abnormal nonspecific blood tests used in neonatal sepsis diagnosis in the national guideline. [file 5790261.f1.zip › Supplementary Table 1.pdf]

Supplemental Table 1. Common infection-related clinical manifestations mentioned in the national guideline.

General condition:temperature instability (fever or hypothermia),feeding intolerance,lethargy or hypotonia
Digestive system:abdominal distension,jaundice,gastric retention,hepatosplenomegaly
Respiratory system:new or increased apnea or respiratory distress,increased requirement on respiratory support
Circulatory system:poor perfusion (mottled skin, prolonged peripheral refill time ≥3s,cold limbs), tachycardia,bradycardia
Urinary system:oliguria or acute renal failure
Hematologic System:hemorrhage or purpura
