# Supplementary material for: Heterogeneity of Neutrophils and Immunological Function in Neonatal Sepsis: Analysis of Molecular Subtypes Based on Hypoxia–Glycolysis–Lactylation
Source: Mediators Inflamm. 2025 Mar 26;2025:5790261. doi: 10.1155/mi/5790261 (PMC11964727; doi:10.1155/mi/5790261)
Supplement: Supporting Information — Figure S1. Normalized box plots of the GSE69686 dataset samples and differentially expressed genes (DEGs). Table S1. Common infection-related clinical manifestations mentioned in the national guideline. Table S2. Abnormal nonspecific blood tests used in neonatal sepsis diagnosis in the national guideline. [file 5790261.f1.zip › Supplementary Table 2.pdf]

Supplemental Table 2. Abnormal non-specific blood tests used in neonatal sepsis diagnosis in the national guideline .

White blood cell count: between 6 hours to 3 days after birth: $\geq 30\times 10^{9}$ /L，$\geq$3 days after birth:$\geq 20\times 10^{9}$ /L,<br>Or $<5\times 10^{9}$ /L at anytime
Immature / total neutrophil，I/T:0 to 3 days after birth:I/T$\geq$0.16; $\geq$3 days after birth:I/T$\geq$0.12
C-reactive protein(CRP): Within 6 hour after birth:CRP$\geq$3 mg/L，6~24hours after birth:CRP$\geq$5 mg/L，>24hours after birth:CRP $\geq$10 mg/L
Procalcitonin (PCT)level:⩾0.5 mg/l (physiological elevation during the first 3 days should be considered).
Platelet: $<150\times 10^{9}$ /L at anytime
