# Supplementary figures and images for: Heterogeneity of Neutrophils and Immunological Function in Neonatal Sepsis: Analysis of Molecular Subtypes Based on Hypoxia–Glycolysis–Lactylation
Source: Mediators Inflamm. 2025 Mar 26;2025:5790261. doi: 10.1155/mi/5790261 (PMC11964727; doi:10.1155/mi/5790261)

A

GSE69686

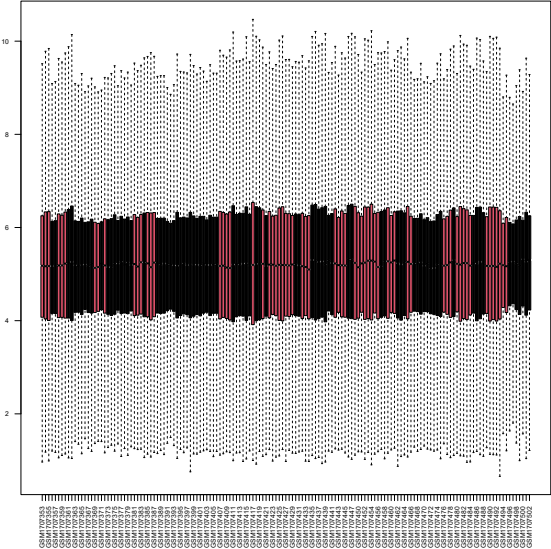

B

GSE69686

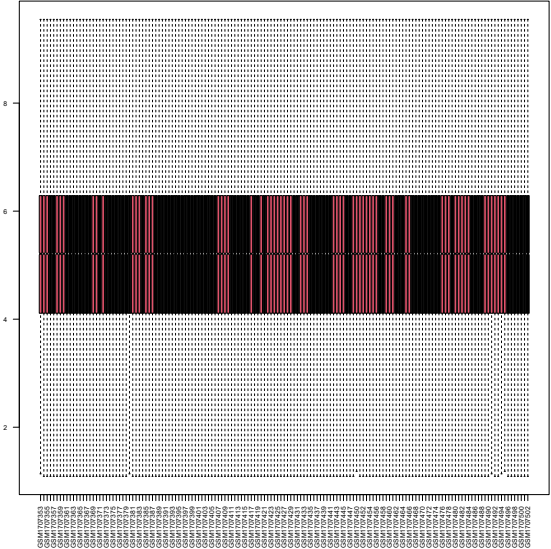

Supplement: Supporting Information — Figure S1. Normalized box plots of the GSE69686 dataset samples and differentially expressed genes (DEGs). Table S1. Common infection-related clinical manifestations mentioned in the national guideline. Table S2. Abnormal nonspecific blood tests used in neonatal sepsis diagnosis in the national guideline. [file 5790261.f1.zip › Supplementary Figure 1.pdf]
